# 

**Published:** 1908-12-15

**Authors:** 


### ANNOUNCEMENTS.

THE NATIONAL DENTAL ASSOCIATION.—The Thirteenth Annual Session of the National Dental Association will be held in Birmingham, Ala., commencing on the last Tuesday in March, 1909, and the following officers of Sections have been appointed:

Section I. H. E. Kelsey, Chairman, Commonwealth Bank Building, Baltimore, Md.; H. H. Johnson, Vice-Chairman, 306 Second Street, Macon, Ga.: J. S. Spurgeon, Secretary, Hillsboro, N. C. Prosthetic Dentistry, Crown and Bridge Work, Orthodontia, Metallurgy, Chemistry and allied subjects.

Section II. W. G. Ebersole, Chairman, 800 Schofield Building, Cleveland, Ohio; R. H. Walker, Vice-Chairman, 231 Main Street, Norfolk, Va.; L. L. Barber, Secretary, 311 Summit Avenue, Toledo, Ohio. Operative Dentistry, Nomenclature, Literature, Dental Education and allied subjects.

Section III. Charles C. Allen, Chairman, Rialto Building, Kansas City, Mo.; J. E. Chase, Vice-Chairman, Ocala, Fla.; J. W. Hull, Secretary, Altman Building, Kansas City, Mo. Oral Surgery, Anatomy, Physiology, Histology, Pathology, Etiology, Hygiene, Prophylaxis, Materia Medica and allied subjects.

V. E. TURNER, *President*,
Raleigh, N. C.

C. S. BUTLER, *Secretary*,
Buffalo, N. Y.

INDIANA STATE BOARD OF EXAMINERS.—This Board will meet in Indianapolis, January 11, 1909. Address for full particulars Dr. F. R. Henshaw, Middletown, Ind.

ORAL HYGIENE IN THE ARMY.—The following circular indicates the earnestness with which the army dental corps is endeavoring to care for the health of our soldiers in the Phillipines.

POST OF ZAMBOANGA.

ZAMBOANGA, MINDANAO, P. I.,

CIRCULAR *August* 27, 1908.

The following recommendations of the Dental Surgeon in reference to the care of teeth are published for the information of all concerned:

"Each man should be provided with tooth brush, tooth powder (Lyons' tooth powder, sold in Commissary, very good), and listerine, one bottle.

"The teeth should be cleaned after each meal; especially is this necessary after the evening meal. If the teeth are neglected at night the particles of food from the last meal remain around the teeth for twelve hours, decaying and becoming putrid.

PROPER WAY TO BRUSH TEETH.

"Wet the brush in water, put plenty of tooth powder on the brush, then brush the teeth upward and downward, using a rotary motion so that the bristles will get in between the teeth. After the teeth have been cleaned, rinse the mouth with equal parts of listerine and water. If listerine is not obtainable, use water alone.

*"CLEAN TEETH DON'T DECAY."*

This circular will be read to the enlisted men of each organization at the first retreat formation after its receipt.

BY ORDER OF CAPTAIN SEAY:

H. A. DRUM,

*Captain and Adjutant, 23d Infantry,*

*Adjutant.*

---

DENTAL COLLEGE GRADUATES, 1908.—Birmingham Dental College, 15; University of California, 20; San Francisco College of Physicians and Surgeons, 18; University of Southern California, 32; Colorado College of Dental Surgery, 13; Howard University Dental College, 5; Georgetown University, 3; George Washington, University, 19; Southern Dental College, 55; Atlanta Dental College, 82; Chicago College of Dental Surgery, 68; Northwestern University Dental School, 155; University of Illinois, 29; Indiana Dental College, 49; University of Iowa, 53; Keokuk Dental College, 18; Louisville College of Dentistry, 61; New Orleans College of Dentistry, 34; Baltimore College of Dental Surgery, 43; University of Maryland, 47; Baltimore Medical College, Dental Department, 17; Tufts College Dental School, 62; Harvard University, 18; University of Michigan, 54; Detroit College of Medicine, Dental Department, 28; University of Minnesota, 40; Kansas City Dental College, 30; Washington University, 47; Western Dental College, 50; St. Louis Dental College, 32; Barnes University, 8; Lincoln Dental College, 15; Creighton University Dental College, 29; New York College of Dentistry, 61; University of Buffalo, 61; College of Dental and Oral Surgery, of New York, 41; Ohio College of Dental Surgery, 40; Western Reserve University, 24; Cincinnati College of Dental Surgery, 13; Starling-Ohio Medical College, Department of Dentistry, 33; North Pacific College of Dentistry, 39; Pennsylvania College of Dental Surgery, 61; Philadelphia Dental College, 74; University of Pittsburgh, 36; University of Pennsylvania, 103; Medico-Chirurgical College, 33; Vanderbilt University, 58; University of Tennessee, 18; Meharry Dental College, 23; The State Dental College, 10; Texas Dental College, 10; University College of Medicine, 13; Marquette University, 23; Wisconsin College of Physicians and Surgeons, 3; Royal College of Dental Surgeons of Ontario, 37; Laval University, 00. Total, 2,061.—*Western Dental Journal.*

---

DISTRICT OF COLUMBIA BOARD OF EXAMINERS.—This Board will meet in Washington City, January 4, 5 and 6,

1909, at Georgetown University. The fee for examination is $10.00 and should be sent to Dr. Starr Parsons, 1309 L Street, N. W., Washington, D. C., not later than December 22, 1908.

